# Predicting the artificial immunity induced by RUTI® vaccine against tuberculosis using universal immune system simulator (UISS)

**DOI:** 10.1186/s12859-019-3045-5

**Published:** 2019-12-10

**Authors:** Marzio Pennisi, Giulia Russo, Giuseppe Sgroi, Angela Bonaccorso, Giuseppe Alessandro Parasiliti Palumbo, Epifanio Fichera, Dipendra Kumar Mitra, Kenneth B. Walker, Pere-Joan Cardona, Merce Amat, Marco Viceconti, Francesco Pappalardo

**Affiliations:** 1 Department of Mathematics and Computer Science, University of Catania, 95125 Catania, Italy; 2 Department of Drug Sciences, University of Catania, Italy, 95125 Catania, Italy; 3 Etna Biotech S.r.l., 95121 Catania, Italy; 4 Department of Transplant Immunology and Immunogenetics, All India Institute of Medical Sciences, New Delhi, 110029 India; 5 TuBerculosis Vaccine Initiative (TBVI), Lelystad, 8219 The Netherlands; 6 Archivel Farma, S.L, 08916 Badalona, Spain; 7 Experimental Tuberculosis Unit (UTE), Fundació Institut Germans Trias i Pujol (IGTP), Universitat Autònoma de Barcelona (UAB), Badalona, Spain; 8 Centro de Investigación Biomédica en Red (CIBER) de Enfermedades Respiratorias, Madrid, Spain; 9 Department of Industrial Engineering, Alma Mater Studiorum, University of Bologna, 40136 Bologna, Italy

**Keywords:** Computational modeling framework, In silico clinical trials, Tuberculosis, Vaccine, Immunity, Therapeutic strategies

## Abstract

**Background:**

Tuberculosis (TB) represents a worldwide cause of mortality (it infects one third of the world’s population) affecting mostly developing countries, including India, and recently also developed ones due to the increased mobility of the world population and the evolution of different new bacterial strains capable to provoke multi-drug resistance phenomena. Currently, antitubercular drugs are unable to eradicate subpopulations of *Mycobacterium tuberculosis* (MTB) bacilli and therapeutic vaccinations have been postulated to overcome some of the critical issues related to the increase of drug-resistant forms and the difficult clinical and public health management of tuberculosis patients. The Horizon 2020 EC funded project “In Silico Trial for Tuberculosis Vaccine Development” (STriTuVaD) to support the identification of new therapeutic interventions against tuberculosis through novel in silico modelling of human immune responses to disease and vaccines, thereby drastically reduce the cost of clinical trials in this critical sector of public healthcare.

**Results:**

We present the application of the Universal Immune System Simulator (UISS) computational modeling infrastructure as a disease model for TB. The model is capable to simulate the main features and dynamics of the immune system activities i.e., the artificial immunity induced by RUTI® vaccine, a polyantigenic liposomal therapeutic vaccine made of fragments of *Mycobacterium tuberculosis* cells (FCMtb). Based on the available data coming from phase II Clinical Trial in subjects with latent tuberculosis infection treated with RUTI® and isoniazid, we generated simulation scenarios through validated data in order to tune UISS accordingly to STriTuVaD objectives. The first case simulates the establishment of MTB latent chronic infection with some typical granuloma formation; the second scenario deals with a reactivation phase during latent chronic infection; the third represents the latent chronic disease infection scenario during RUTI® vaccine administration.

**Conclusions:**

The application of this computational modeling strategy helpfully contributes to simulate those mechanisms involved in the early stages and in the progression of tuberculosis infection and to predict how specific therapeutical strategies will act in this scenario. In view of these results, UISS owns the capacity to open the door for a prompt integration of in silico methods within the pipeline of clinical trials, supporting and guiding the testing of treatments in patients affected by tuberculosis.

## Background

Tuberculosis (TB) is a transmissible airborne disease triggered by the *Mycobacterium tuberculosis* (MTB) acid-fast bacillus that most often affect the lungs [1, 2]. Even though TB has existed for millennia, it still represents a worldwide health problem and one of the major causes of morbidity and mortality in developing and developed countries. In 2016, 10.4 million of new cases and 1.6 million of TB-causing deaths were globally estimated; of these, 2.79 million were new cases in India and 435,000 cases dead for TB [3]. For these reasons, India is the country with the highest burden of TB and leads the world in deaths from tuberculosis in terms of absolute number of cases [4]. TB is spread through the air from person to person, in particular when people with lung TB cough, sneeze or spit. The lifetime risk of falling ill with TB for people previously infected with TB bacilli is about 5–15%. However, compromised immune systems subjects, such as people living with diabetes, HIV or malnutrition conditions have a much higher risk of falling ill with TB [5–9].

For an organism encountering M. tuberculosis bacilli there are different possible outcomes: firstly, the bacillus can be instantly killed by the host’s innate immune response [10, 11]. Secondly, of every 10 people infected with M. tuberculosis, one may develop an active infection in their lifetime within a finite time frame, from 1 to 3 years. This category probably lacks the capability to both control the early infection and develop a protective response in time in order to prevent the disease. Cough with sputum and blood at times, fever, weakness, chest pains, weight loss and night sweats are the major common symptoms of active pulmonary TB [12]. Finally, we observe latent tuberculosis infection (LTBI) when a persistent immune response to stimulation by *Mycobacterium tuberculosis* antigens occurs without evidence of clinically manifested active tuberculosis. One-quarter of the global population is infected with LTBI and individuals with LTBI represent a reservoir for active TB cases [13, 14].

In order to manage the disease, antibiotic treatment reduces the bacterial load in the lungs and can be helpful to reduce the probability of transmission, along with other public health measures [15]. The anti-tuberculosis drugs can be classified as drugs with bactericidal and sterilizing effect. The first one is fundamental in the early phase giving a significant reduction of the bacterial load; the second one is more efficient in the continuation phase, because it is directed to kill latent mycobacteria. The secondary consequence of this activity is the decrease of the probability of selecting drug-resistant strains [16].

The mainstay of TB antibiotics consists on a first-line therapy for active tuberculosis. The standard treatment for people at low risk for drug-resistance is isoniazid (INH), rifampicin (RMP), pyrazinamide (PZA) and ethambutol (EMB) or streptomycin (SM) for the initial two-month phase followed by isoniazid and rifampin for 4 months to 7. The main properties of these anti-tuberculosis drugs are the bactericidal and sterilizing activity along with a reduction in the development of drug resistance [17, 18].

Second line drugs are used for the treatment of drug resistant TB patients (i.e., for single or multidrug-resistant tuberculosis (MDR-TB) as well as extensively drug-resistant tuberculosis (XDR-TB)) or and the World Health Organization (WHO) has divided them in 2, 3 and 4 group [19]. Aminoglycosides and polypeptides belong to WHO group 2 (i.e., amikacin (AMK) and capreomycin); fluoroquinolones are attributed to WHO group 3 such as ciprofloxacin (CIP), levofloxacin (LVX), moxifloxacin (MXF) while thioamides and cycloserine are representative drugs of WHO group 4 for example with ethionamide [20, 21]. Currently treatment regimens vary in duration between 6 months in drug susceptible TB to 24 months in drug resistant TB [22].

Rifamycin-based regimens for LTBI have been successful in preventing progression to TB disease [23]. However, treatment of LTBI needs a long period of chemotherapy, approximately 9 months, leading to an extremely difficulty in terms of management and treatment-compliance therapy [24].

The lengthy TB chemotherapy regimen likely reflects the inability of current chemotherapy drugs to eradicate subpopulations of Mycobacterium bacilli, allowing these population bacteria to persist in infected individuals, surviving in granulomas with a central necrotic core and an external layer of foamy macrophages (FM) as the main immunosuppressive barrier [25].

Moreover, the dramatic changes from an epidemiological point of view during the last two decades and the increase of drug-resistant forms of tuberculosis considerably complicate the clinical and public health management of the patients and of their relative contacts [26].

Therapeutic vaccinations have been suggested to overcome these critical issues and several candidates are already in clinical phases of development [27]. In this regard, efforts to shorten treatment duration have been successful, as shown in recent clinical trials, evaluating the role of several immunotherapeutic vaccines, such as RUTI® [28, 29]. The safety and immunogenicity of RUTI® vaccine have been already tested in Phase I and II studies in healthy volunteers and LTBI infected patients and it was observed its capacity to stimulate host immune effectors against bacilli improving outcomes under chemotherapy treatment [30].

Inside the “In Silico Trial for Tuberculosis Vaccine Development” (STriTuVaD) project, the anti-TB vaccine RUTI® is under evaluation in under evaluation in studies reducing the time to negativity of Sputum Culture Conversion (SCC) [31] in TB drug sensitive patients and TB multidrug resistance patients, favorably responding to standard TB treatment, with the aim to shorten treatment and reduce the frequency of recurrence. In this context, the application of in silico computational modeling strategies in the pipeline of standard clinical trials can helpfully increase the high likelihood chance of success of promising vaccine through the simulation of each individual of the reference population and the in silico predictions in terms of vaccine efficacy, recurrence and reactivation of the disease [32–34].

Within the STriTuVaD consortium, the Universal Immune System Simulator (UISS) platform (previously successfully applied to a large number of disease modeling scenarios [35–42]) simulates the MTB infection dynamics and its interactions with the host immune system. Hence, the in silico trial platform will be delivered to the STriTuVaD multidisciplinary consortium in order to simulate the relevant individual human physiology and physiopathology in patients affected by *Mycobacterium tuberculosis* and to predict how specific vaccinations strategies will be effective or not.

Here, we present the current advances of UISS development and its extension in the context of tuberculosis [43], focusing on specific scenarios that show the capability of UISS in simulating the intrinsic immune system behavior against MTB infection (eliciting or not eliciting the complete clearance of the infection or, eventually, allowing the chronic establishment of MTB reservoir inside the host due to both MTB characteristics and genetic features of the host).

Based on the available data coming from phase II Clinical Trial in subjects with latent tuberculosis infection treated with RUTI® [29] and then with 1 month of isoniazid treatment, we analyzed three simulation scenarios in order to tune UISS through previous validated data and accordingly to STriTuVaD objectives.

The first scenario simulates the establishment of MTB latent chronic infection with some typical granuloma formation as a reservoir of MTB infection; the second scenario deals with a reactivation phase during latent chronic infection; the third represents the latent chronic disease infection scenario during RUTI® vaccine administration in order to enhance antibiotic therapy.

## Implementation

### The simulation framework

UISS has a long history of development. It branches from C-IMMSIM [44] to specialize the simulation framework to deal with tumor immunology. Then it held different stages of immune system features development and was applied to a wide disease modeling scenarios. Nowadays, a lot of computational efforts are leading to an incredible boost in biomedicine research [45, 46]. Technically speaking, UISS is an agent based model (ABM) [47]. It can be thought as a two-component software. The first one is represented by a core that implements all the immune system features that is continuously updated to take into account all the state-of-the-art advancements in immunology. It owns anatomical compartments, cells and molecules, immune system repertoire, molecular affinity, haematopoiesis with generation of cells, cell maturation and thymus selection, Hayflick limit in the number of duplications [48], aging and memory of past infections, hyper-mutation of antibodies [49], bystander effect [50], cell activation and anergy, cell interaction and cooperation, antigen digestion and presentation. From the point of the view of physical representation, UISS maps biological tissues, lymphatic system and peripheral blood into a mathematical structure called cartesian lattice (two or three dimensional). Thymus and bone marrow are taken into account in an implicit way. This means that positive and negative selection (that are the main features of thymus) are implemented to educate T cells. A procedure that generates mature B cells, instead, models the bone morrow cells production. UISS playground is then only populated by immunocompetent entities. An important working assumption that allows UISS to deal with the incredible and fascinating variability of the immune system repertoire (including its capacity to adapt and consequently mount immune response) is the stochastic bitstring polyclonality. This means that epitopes and paratopes are modeled using binary calculus. Each of them is represented by a set of binary strings. So, having *N* bits allow a potential immune system repertoire diversity of 2^N^. Stochasticity is implemented as probabilistic rules that produces a logical causal/effect sequence of events culminating in the immune response and development of immunological memory.

The second part consists on the disease model. It encapsulates all the characteristics that are needed to reproduce the biological scenario one would like to simulate. In this specific case, the disease model takes into account the entities, the interactions and the dynamics of MTB against its natural target and with the immune system of the host.

To simulate the effects of RUTI® vaccination strategy, we put into the model the liposome dynamics and how it interacts with the immune system. In particular we extended UISS framework to include: activation of PRRs that triggers the initiation of the innate immune response; activated CTLs that recognize peptides bound to the major histocompatibility complex class I and II molecules (MHC-I, MHC-II), which express antigenic peptides on APCs and bind to T cells via the T-cell receptor. TH cells provide help to antigen-specific B cells, resulting in antibody production.

### The disease conceptual model

To model the entire dynamics of MTB and interactions with the immune system machinery, we selected all the players that have a role in TB in order to include the disease model into the simulation framework. The starting point was the implementation of all the biological entities involved into the dynamics of MTB, both at cellular and molecular scale. To analyze the behavior of a possible therapeutic intervention, we selected one of the vaccines we are going to test inside the STriTuVaD project i.e., RUTI® vaccine. To this end, we implemented inside UISS the main mechanism of action (MoA) of this immunotherapeutic vaccine.

**Fig. 1:**
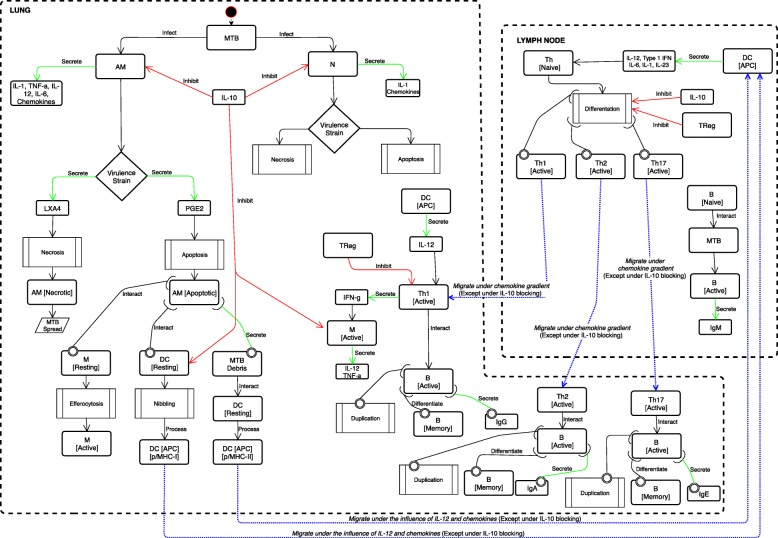
The pulmonary tuberculosis – immune system interaction disease model. Conceptual description of the main entities and interactions of MTB – immune system. The main two compartments are represented: the lung and the peripheral lymph nodes. The representation depicts both cellular and humoral response when MTB droplets infect alveolar macrophages resident in the lung. The cascade of cytokines and chemokines is also represented with possible different behaviors depending on the virulence of the MTB strain

Our model takes into account both innate and adaptive immunity (both cellular and humoral) and immune memory. Figure 1 depicts all the entities implemented within the simulation framework, especially immune cells, cytokines and specific biological processes along with their peculiar properties in TB dynamics. We used Unified Modeling Language (UML) to design the conceptual model. UML is a general-purpose modeling language that is extensively used in the field of software engineering to provide a standard way to visualize the design of a system. All the entities of the TB disease model interact each other, and are appropriately located in two specific compartments: the lung (pulmonary alveoli) and the peripheral lymph nodes.

The starting point of our TB disease model consists on the aerosol droplets of MTB that reach lung alveolar macrophages (AMs) on one side and neutrophils (N) on the other one. The initial challenge of MTB is implemented simulating a virtual injection of MTB bacilli into a lattice point in the lung compartment. Then the bacilli are free to move and disseminate randomly. When an AM becomes infected, it secretes IL-1, TNF-α, IL-12, IL-6 and chemokines. Depending on MTB strains and their virulence, infected AM can play a different role in determining the downstream pathways leading to the induction of either apoptosis or necrosis and to the final outcome of the infection. In this context, lipoxin A4 (LXA4) promotes necrosis, while prostaglandin E2 (PGE2) is a proapoptotic factor. When necrosis process is favored, the AM becomes necrotic and contributes to the MTB spread. Otherwise, when the AM becomes apoptotic, simultaneously three specific scenarios can occur. Firstly, AM apoptotic can interact with a lung resting macrophage (M) and lead to efferocytosis of macrophages, in other words an engulfment of AM apoptotic by M, essential for tissue homeostasis and immunity. This means switches from “resting” to “active” status.

Secondly, AM apoptotic cells can encounter a lung dendritic cell (DC). AM apoptotic can be taken up by DC that capture antigens (Ag) through a process called nibbling; then, DC will process and present the resulting fragments to antigen-specific T lymphocytes in the context of molecules of the major histocompatibility complex of class I (MHC-I) or related proteins. From this point forward, MTB–antigen processing DCs, migrate to the local lung-draining lymph nodes (by 8–12 days post infection) driving naïve T cell polarization. This migration is influenced by IL-2 release and other chemokines, except when IL-10 is present and is able to block this moving. A third interaction dealt with the secretion of MTB debris from AM apoptotic: MTB debris will interact with DCs, in status resting, that will process and present the resulting fragments to antigen-specific T lymphocytes in the context of molecules of the major histocompatibility complex class II (MHC-II) or related proteins.

When MTB infects a lung N, N produces and secretes IL-1 and other chemokines. Just like AM, also for N the MTB strain can lead to a different role in the induction of either apoptosis or necrosis and to the final outcome of the infection.

Both AM and N effector functions, can be negatively modulated by IL-10 induction during MTB infection. Respectively, IL-10 can lead to the inhibition of macrophage and neutrophil effector functions, with reduction of bacterial killing and impairing of secretion of cytokines and chemokines.

As previously said, IL-10 can also block chemotactic factors that control DC moving to the lung-draining lymph nodes.

The scenario inside the lymph node depicts the DC cells in antigen presenting cell status secreting IL-12, Type 1 IFN, IL-6 and IL-23 and driving naïve T cell differentiation toward a Th1, Th2 or Th17 phenotype. Th cell population differentiation can be negatively modulated by IL-10 and regulatory T cells (TReg). Protective antigen-specific Th1 cells migrate back to the lungs about 14–17 days after the initial exposure and infection to MTB and under a chemokine gradient (except when IL-10 blocks this process). In the lung, activated Th1 cell population, produce and secrete IFN-γ, causing macrophage activation, relative cytokine production (IL-12 and TNF-α) and bacterial control. It is worth to mention that in this context, IL-10 can block macrophage activation and consequent cytokine secretion. Also TReg negatively modulate Th1 population effector functions.

For the sake of completeness, the Th1 cell population interacts also with B cells leading to three specific processes at the same time: after a successful interaction, B cells duplicate, differentiate in memory B and secrete immunoglobulins type G (IgG).

Similarly, Th2 cell migration and interaction with B cells leads to B cells duplication, differentiation in memory B cells and secretion of immunoglobulins type A (IgA). For what concern Th17 cell population, their migration and interaction with B cells will finally lead to cell duplication and secretion of immunoglobulins type E (IgE). Inside the lymph node, B cells interact also with MTB; after that B cells become active and secrete immunoglobulins type M (IgM).

## Results and discussion

With the aim to obtain accurate in silico predictions about the efficacy of therapeutical interventions directed against MTB, we run different simulations to evaluate the degree of precision of UISS when applied to a specific interventional vaccine scenario based on a promising vaccine i.e., RUTI® vaccine. We used publicly available data coming from phase II Clinical Trial in subjects with latent tuberculosis infection treated with RUTI® [29], to evaluate the immunogenicity. We analyzed three simulation scenarios. The first one is represented by the establishment of MTB latent chronic infection with some typical granuloma formation as a reservoir of MTB infection. The second one deals with a reactivation phase during latent chronic infection where a possible breakdown of the granuloma may lead to the spread of the bacilli and to the reactivation of the disease, with an increased necrotic burden. The third consists on the latent chronic disease infection scenario during RUTI® vaccine administration: here we show the evolution in time of M and CD4 T cell populations, in conjunction with the temporal evolution of IFN-γ that owns an important role as a biomarker of protection.

**Fig. 2:**
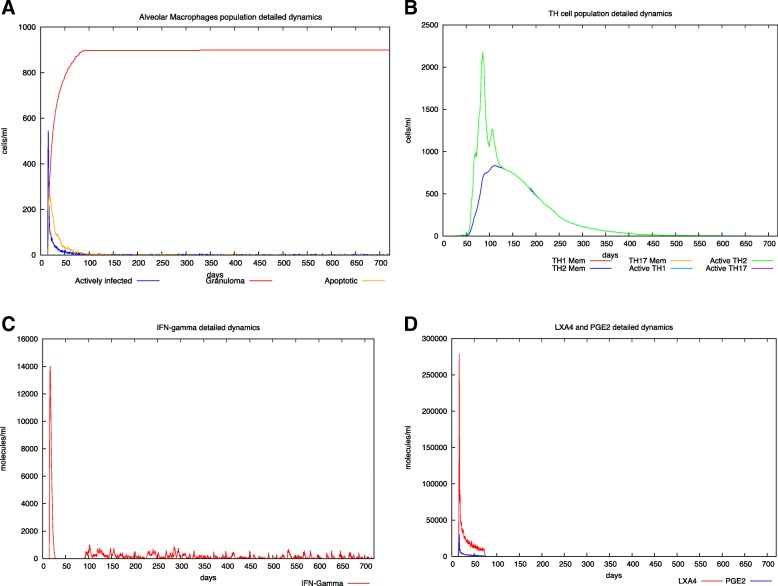
In silico latent tuberculosis infection scenario. Panel A depicts AM population dynamics. In particular, it is simulated the time course of the MTB-alveolar macrophages infected levels, the apoptotic ones and those forming granuloma. One can appreciate the typical granuloma behavior in LTBI as a potential protective immune system instrument from one side and the MTB reservoir of infection from the other one. Panel B shows Helper T cells dynamics. Subtypes (TH-1, TH-2 and TH- 17) are reported. LTBI induces a TH-2 switch instead of TH-1. This leads to a lesser immune system activation that could overcome the MTB spread. Panel C describes IFN-γ dynamics. After the first spike (representing the initial inflammatory response soon after the MTB infection), IFN-γ levels are kept almost at low level, indicating a latent typical scenario of immune system tolerance. In panel D LXA4 and PGE2 detailed dynamics are reported. The simulator correctly predicts a predominant LXA4 level indicating a pro-necrotic induction commonly observed in virulent strain of MTB causing a LTBI scenario. For all the simulated scenarios, time has been set to 720 days (2 years) and the virtual patient has been challenged with MTB at day 40

In Fig. 2 panel A, an initial infection of a virulent strain of MTB is supposed to happen at day 40. MTB rapidly infects both AM and M that become infected (blue lines). A similar behavior (not shown here) also occurs for N. Then, MTB grows inside them eventually leading to either apoptosis or necrosis of the infected cell, accordingly to the levels of LXA4 and PGE2 in the site of infection. In particular, the virulent strain will drive towards the production of the pro necrotic LXA4 (panel D) and thus to higher percentages of necrotic cells that will become part of the granuloma mass (red lines). After the formation of the granuloma that represents a reservoir of MTB infection, the infection remains mostly latent, and also the CD4 T response that is recruited at the initial stages of infection (panel B) tends towards baseline levels. This is also present in the Interferon-gamma plots (panel C) where after an initial peak, its level drastically drops.

**Fig. 3:**
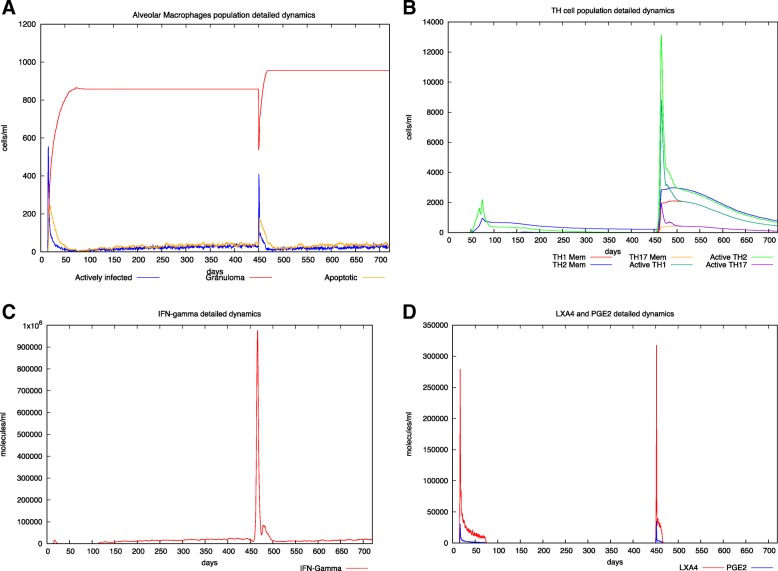
In silico latent tuberculosis infection with reactivation event scenario. Panel A depicts AM population dynamics. In particular, it is simulated the time course of the MTB-alveolar macrophages infected levels, the apoptotic ones and those forming granuloma. One can appreciate the breakdown of the granuloma leading to a successive reactivation at day 450. Moreover, granuloma increases in dimension indicating a worsening of the disease. Panel B shows Helper T cells dynamics. Subtypes (TH-1, TH-2 and TH-17) are reported. At the reactivation time, it is reported an evident TH-2 switch. However also a TH-1 driven response is present. Panel C describes IFN-γ dynamics. The peak of IFN- γ indicates an attempt of immune system to contain the MTB spreading. In panel D LXA4 and PGE2 detailed dynamics are reported. The simulator correctly predicts a predominant second peak of LXA4 indicating a pro-necrotic induction commonly observed in virulent strain of MTB. For all the simulated scenarios, time has been set to 720 days (2 years) and the virtual patient has been challenged with MTB at day 40

In Fig. 3 a breakdown of about the 50% of the total granulomatous burden is supposed to happen approximately after 400 days after primary infection. This translates in a rapid drop in the number of entities that are part of the granuloma (yellow lines) and in a reactivation of the disease with a final worsening of the total granuloma burden, even if an immune response is present (see panels B and C), that is however unable to deal with the spread of the bacteria.

**Fig. 4:**
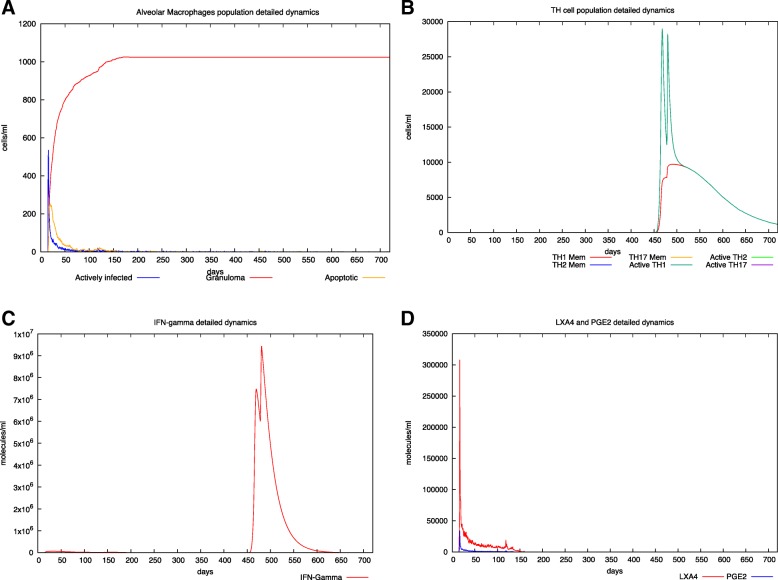
In silico latent tuberculosis infection with RUTI vaccine administration. Panel A depicts AM population dynamics. In particular, it is simulated the time course of the MTB-alveolar macrophages infected levels, the apoptotic ones and those forming granuloma. Panel B shows Helper T cells dynamics. Subtypes (TH-1, TH-2 and TH-17) are reported. The RUTI vaccine has been administered at day 450 and at day 478 (two inoculations, 28 days interval). CD4 T cells (TH-1 subtype) are the predominant one, indicating a strong immune system response induced by the therapeutical intervention. Panel C describes IFN-γ dynamics. Here one can appreciate the presence of two peaks of IFN-γ indicating the vaccine activity in stimulating pro-inflammatory and TH-1 mediated immune system response. Moreover, the level of IFN-γ dynamics prediction mirrors the one observed in the RUTI phase II clinical trial. In panel D LXA4 and PGE2 detailed dynamics are reported. The simulator correctly predicts a predominant peak of LXA4 indicating a pro-necrotic induction commonly observed in virulent strain of MTB. For all the simulated scenarios, time has been set to 720 days (2 years) and the virtual patient has been challenged with MTB at day 40

Finally, in Fig. 4 we show the effects of the vaccination with the RUTI® vaccine in an individual with latent TB infection. Even if the vaccine seems to be mostly ineffective against the existing granuloma burden (panel A), a strong Th1 induced response is elicited with the induction of immunological memory (panel B). High levels of IFN-γ are present, in good agreement with the results presented in [29] for a 25μg dosage of the RUTI® vaccine in latent patients.

Overall, the simulations results are in very good agreement with available data coming from clinical trials. In particular, the in silico framework shows reliability in capturing the main landmarks of both induced immune response mirroring the IFN-γ, CD4 T cells, LXA4 and PGE2 dynamics. Moreover, form the spatial point of view, the granuloma dynamics with MTB spread were appropriately reproduced during the two most widespread scenarios characterizing pulmonary tuberculosis i.e., latent and latent with reactivation.

## Conclusions

Computational models are important for the understanding of biological systems. Such models can be applied to enhance or predict therapeutic effects at the organism level. The pharmaceutical companies suggest that computational biology can play an excellent role in this field. In silico models can afford answers to the general behaviour of the immune system, the analysis of cellular and molecular interactions, the effects of treatments, and the course of diseases. The main goal of the STriTuVaD project is to create a computational infrastructure able to predict the general outcome of two different vaccination strategies against M. tuberculosis. In this work, we presented UISS framework implementation able to simulate the artificial immune response elicited by the vaccines. In particular we implemented the components of RUTI® vaccine.

Further steps deal with the creation of a set of virtual subjects with the aim to reproduce biological diversity of the subjects we will simulate.

Through a “vector of features” that combines both biological and pathophysiological parameters we will obtain a personalization of the virtual patient to reproduce the physiology and the pathophysiology of the subject.

This UISS simulation platform will be able, when compared to the experimental results of the clinical trial phase II, to predict with the necessary accuracy the bacterial load in the sputum smear.

## Availability and requirements

Project name: UISS for TB.

Project home page: https://www.combine-group.org/software

Operating system(s): Platform independent.

Programming language: C and Python.

Other requirements: none.

Any restrictions to use by non-academics: not applicable.

## Data Availability

The main computational framework is fully described in the paper.

## Abbreviations

ABM: Agent Based Modeling
Ag: Antigens
AM: Alveolar Macrophage
AMK: Amikacin
APC: Antigen Processing Cell
CIP: Ciprofloxacin
DC: Dendritic Cell
EMB: Ethambutol
FCMtb: Fragments of *Mycobacterium tuberculosis* cells
FM: Foamy macrophages
HLA: Human Leukocyte Antigen
IgA: Immunoglobulins type A
IgE: Immunoglobulins type E
IgG: Immunoglobulin class G
IgG: Immunoglobulins type G
IgM: Immunoglobulins type M
INH: Isoniazid
LTBI: Latent Tuberculosis Infection
LVX: Levofloxacin
LXA4: Lipoxin A4
M: Macrophage
MDR-TB: Multidrug-resistant Tuberculosis
MHC-I: Major Histocompatibility Complex class I
MHC-II: Major Histocompatibility Complex class II
MoA: Mechanism of Action
MTB: *Mycobacterium tuberculosis*
MXF: Moxifloxacin
N: Neutrophils
PGE2: prostaglandin E2
PZA: Pyrazinamide
RMP: Rifampicin
SCC: Sputum Culture Conversion
SM: Streptomycin
STriTuVaD: In Silico Trial for Tuberculosis Vaccine Development
TB: Tuberculosis
TReg: Regulatory T cells
UISS: Universal Immune System Simulator
WHO: World Health Organization
XDR-TB: Extensively Drug-resistant Tuberculosis
